# The role of albumin in compound transport: new possibilities by intravital imaging

**DOI:** 10.17179/excli2022-5641

**Published:** 2022-12-08

**Authors:** Amira Hamdy

**Affiliations:** 1 Forensic Medicine and Toxicology Department, Faculty of Veterinary Medicine, South Valley University, Qena, Egypt

## ⁯

Albumin is the most abundant plasma protein with a concentration of 3.5-5 grams per deciliter (Merlot et al., 2014[13]). It plays a critical role in pharmacokinetics/toxicokinetics of both endogenous molecules and xenobiotics (Kratz and Elsadek, 2012[11]). Since concentrations of albumin in the blood can be strongly reduced in patients with liver or kidney diseases (Balcar et al., 2021[1]; Campos et al., 2020[2]; Haller, 2005[5]; Hartl et al., 2021[6]; Holland et al., 2022[9]), transport of xenobiotics and endogenous molecules may be critically altered under these conditions. However, the influence of hypoalbuminemia on the transport and toxicity of xenobiotics is not fully understood. This is due to the lack of techniques that allow direct visualization of xenobiotic transport from blood to a specific tissue compartment. A recent study presented new techniques to analyze the role of albumin in pharmacokinetics by intravital spatio-temporal imaging using the mycotoxin ochratoxin A as an example (Hassan et al., 2022[8]). Ochratoxin A has a very high albumin binding capacity (>99 %) which explains its very long half-life under normal conditions (Malir et al., 2016[12]). Using albumin knockout mice where albumin plasma concentrations in heterozygous mice are reduced to a similar extent as in patients with severe hypoalbuminemia, the authors showed that ochratoxin A is rapidly transported from the blood into hepatocytes and into kidney tubular epithelial cells which massively shortened its half-life in the blood. Consequently, ochratoxin A-induced hepatotoxicity and nephrotoxicity was dramatically aggravated under conditions of hypoalbuminemia. This suggests that serum albumin acts as a sponge that sucks up OTA and only the remaining very low free fraction can be transported to the cells. This new development was made possible using two-photon based intravital imaging techniques with a spatial resolution of approximately 250 nanometers and a temporal resolution in the millisecond range (Ghallab et al., 2021[4], 2022[3]; Hassan, 2016[7]; Remetic et al., 2022[14]; Vartak et al., 2021[15]). The auto fluorescence properties of ochratoxin A (Jiang et al., 2020[10]) allowed label-free intravital imaging.

A limitation of this study is that the role of hypoalbuminemia on compound transport was investigated so far for only one compound, ochratoxin A. It would be interesting to study the role of hypoalbuminemia in the transport of other compounds with high albumin binding capacity and for compounds that require active transport mechanisms. This will allow to further study the concept that the free fraction (not bound to albumin) of a compound determines its hepato- and nephrotoxicity.

## Conflict of interest

Nothing to disclose.
